# Great and Enduring Deeds

**Published:** 1890-01-11

**Authors:** 


### The Hospital

A Weekly Journal of

Science, Medicine, Nursing, and Philanthropy.

*For Hospitals, Asylums, Medical Practitioners, Students, Nurses, Families, Parishes, Congregations, and the Charitable Public.*

Vol. VII. No. 172.] [January 11, 1890.

### GREAT AND ENDURING DEEDS.

Science, as represented by Mr. Darwin, affirms that the highest type of man is he who is best endowed with moral and religious sentiments. Intellect by itself never produces the most valuable results for the tribe or the nation. It is only when mental capacity is associated with an irresistible sense of duty to fellow-man, and an overwhelming conviction that a just and benignant Deity is to be venerated and served at all costs, that the noblest ideal of human creatures stands revealed to his contemporaries. These propositions are not advanced from a religious, but from a scientific point of view. The physician who is also the physiologist cannot refrain from striving to take measure of the progress of man through the ages. Is it an actual and positive certainty that in a remote past the ancestors of man were masses of undifferentiated protoplasm, exactly as amoebiform creatures are at the present day? Is it true in a hard and tangible sense that at a later stage they were passing through an Ascidian experience? Ages afterwards had they reached the Simian type, and did they know hunger and thirst, passion, and desire, uncontrolled by reason or a moral sense? And are we, their descendants, of the same race as the Shakespeares and the Miltons, the Newtons and the Washingtons, the Howards and the Lincolns, the Darwins, and the Lightfoots? Is all this in sober truth verified and verifiable science, so that, reasoning from the past, we may inspire and guide the present, and with certainty predict the future?

These are eminently practical questions, at any rate for the scientist who has the smallest particle of living faith in his own hypotheses and arguments. But they do not merely affect the scientist and his reasonings; they have the most important ethical bearing on the immediate present, both for the leading spirits of the times and for the private citizen. Whether of the two is the higher way of life from the scientific standpoint—the way of the man who lives for the gratification of his own peculiar impulses and individuality, or the way of him who, seeking the purest and clearest moral light he can find, devotes himself and all his powers and belongings to the following of that pathway which the pure moral light most clearly indicates? This question, which religion answered four thousand years ago, and reaffirmed two thousand years later, science asks to-day. And science, in the person of the late Mr. Darwin, has given the same answer as religion.

These considerations are suggested by certain munificent gifts conferred upon the public during the past few months, and more especially by the offer made by an anonymous donor, through Sir William Scovell Savory, of a hundred thousand pounds, for the founding of a Convalescent Home. There are not wanting persons innumerable who will see in this and other noble gifts to the public merely an attempt on the part of the donors at self-exaltation and self-advertisement. It is probably as true as anything can be that those who see in a good deed a display of selfishness under the guise of benevolence are at heart entirely infamous. The man who does not believe any good of others has first found himself empty of all good. But if he were well advised he would at least keep silence, and not proclaim to all the world that, measured by the standard of science, he is on the level of those mindless creatures that have not yet developed benevolence and a moral faculty. We are glad for our part that this latest public benefactor has for the present chosen to withhold his name. We can thus express to him our grateful sense of his worth as a citizen and as a public benefactor. It is to be hoped, however, that he will not long hide his identity. What harm can it do to a man to have his good deeds known and approved? But it can do harm to the public to be deprived of the opportunity of expressing its gratitude and sense of obligation to the very person himself to whom it is due. The convalescent home which is to be founded by this anonymous munificence will be an institution of extreme practical importance. It is the barest truth to say that hundreds of persons are compelled to leave hospitals before their cure is completed, because innumerable other sick ones are waiting for their beds. Those too soon dismissed patients, leaving the plentiful comfort of the hospital for the "chill penury" of their depleted homes, too often cease to gain strength, and, falling gradually back into diseased helplessness, have to return to the hospital for a fresh period of treatment and cure. From the point of view of experienced philanthropists, this is a grievous waste of charitable resources, whilst from the standpoint of the unfortunate patient it is a double affliction, and results in permanent impairment of the strength.

The unutilised wealth of Great Britain and her colonies is incalculable. If any clairvoyant could look into the hearts of some of the wealthier inhabitants of this country he would probably find a highly honourable struggle in active progress. Many rich men, and women too, undoubtedly have it in their minds to do large deeds of benevolence for their fellow-men. But they fear the people. They dread the rancorous tongues of cynical and spiteful neighbours. They are afraid to be thought pretentious and self-advertising. The non-wealthy forget that the wealthy are men and women like themselves, and may be timorous and cowardly even in the doing of good deeds. But the wealthy who hate ostentation should not permit the vile spite of the worthless cynic to stay their hands, even for a moment. The piteous cry of the suffering and the trumpet-call of duty should put to silence all other tongues, and alone direct the actions and the life.

Since it is a chief part of our business to point out to readers from time to time the most immediately urgent philanthropic work, we venture to inform them, as a fact that admits of no dispute, that the great hospital necessity of the present hour is more beds for consumptive patients. Consumption in its earlier stages is a curable disease. In its later developments it is absolutely hopeless. It is a disease that attacks youths and maidens at the most joyous time of life. For a young man to be seized by this dread enemy and to know that adequate medical skill and nursing care would probably restore him to health, but that by no means can he procure either the skill or the care, is inexpressibly pathetic. There is no heart that knows how to feel at all but would willingly do something to help to snatch such youths and maidens from an all too early grave. If but ten of the hundreds of wealthy men in these islands would each give a hundred thousand pounds to this object, existing consumption hospitals might be enlarged, and new ones might be built where they are most needed. There is at Mount Vernon, Hampstead, a consumption hospital which stands on one of the healthiest sites in England. It contains a mere score or two of beds. There is ground for the erection of a building of six times the size. If an adequate hospital could be erected at Mount Vernon the consumptives of all parts of London and the country would experience an increase of hope. No words that we could use would be more impressive than the simple fact that hundreds of consumptives die every year, a proportion of whom would undoubtedly be saved if they could be treated at consumption hospitals.

Men follow one another in good works as well as in bad; though too often it seems as if the infection of vice were much more energetic than the contagion of virtue. Yet the examples shown of late by persons of wealth and eminence will certainly bear fruit. The means to do large charities are in many hands, and it cannot but be that in many cases the disposition is there too. What is needed on the part of the wealthy is a stronger impulse of benevolence, and a more resolute and effective will for great, noble, and enduring deeds.

